# Moral distress and perception of futile care in intensive care nurses

**Published:** 2015-02-23

**Authors:** Fariba Borhani, Somayeh Mohammadi, Mostafa Roshanzadeh

**Affiliations:** 1 Associate Professor, Medical Ethics and Law Research Center, Shahid Beheshti University of Medical Sciences, Tehran, Iran;; 2 MSc in Nursing, Shahrekord University of Medical Sciences, Shahrehkord, Ira;; 3 Mentor, Surgical Care Research Centre, Birjand University of Medical Sciences, Birjand, Iran.

**Keywords:** moral distress, futile care, intensive care unit, nursing ethics

## Abstract

Special characteristics of care environments have always presented nurses with some challenges. One particular situation is futile care, which is frequently accompanied by countless moral and legal challenges. The dominant atmosphere in futile care may cause moral distress to nurses and lead to a sense of guilt, pain, suffering, job dissatisfaction, and eventually cause nurses to leave the job. This descriptive-analytical study attempted to investigate the relationship between futile care and moral distress in intensive care nurses. Study subjects were 300 nurses in intensive care units in Kerman, Iran and were selected by convenience sampling based on inclusion criteria. Study tools included Corley’s 21-item questionnaire on moral distress and a researcher-made 17-item questionnaire on futile care. Data analysis was performed using SPSS version 16 and suitable analytical and descriptive tests.

The results showed a significantly positive relationship between moral distress and futile care (*P* = 0.03, r = 0.4). Based on the obtained results, futile care can create conditions that may lead to moral distress in nurses and therefore strategies should be devised to prevent these conditions. Moreover, distress in nurses should be identified by periodical counseling so that it can be managed more efficiently.

## Introduction

Due to its humanistic nature, medicine and in particular the treatment and care process have always entailed moral problems and challenges (1). When a person is aware of the right moral action but feels unable to perform it, he/she experiences moral distress (2). 

When confronting moral distress, people worry about their moral integrity, which leads to increased stress as well as physical disorders like headaches and insomnia, or emotional symptoms like reduced self-confidence, anger and feelings of worthlessness (3). Distress in nurses may have internal manifestations like job insecurity or lack of confidence, or external outcomes like decreased relationships with other health team members, reduced or non-existent organizational support, fear from legal actions, increased distress and its consequent unfavorable effects (4).

Moral distress and its negative impact on the treatment process can have certain undesirable outcomes. Care providers may experience reduced moral sensitivity and become indifferent to ethical issues. Moreover, in order to conquer moral distress, nurses may use different strategies, which can in turn lead to burnout (4). This may cause numerous problems for patients and health organizations. When nurses feel stressed, bored and dissatisfied with their jobs, they may consider quitting, which is clearly harmful for both patients and health care organizations (5). It should be noted that different factors contribute to moral distress such as organizational limitations (human forces or equipment), insufficient care and health service resources, and inappropriate requests and demands of patients and their families (6).

One of the important causes of moral distress is futile care, which refers to a potentially purposeful treatment that has no effect on the quality of a patient’s life. In fact, technological advances in medicine often make it possible to prolong the life of patients who have little hope of recovery (7, 8).

Futile care is associated with numerous moral, legal, and medical challenges. From the moral point of view, futile care stands between usefulness and uselessness (9). On the other hand, health care systems should concentrate on treatment and should not be avoided merely due to poor prognosis (10). In terms of legal issues, everyone has a right to make decisions about their health and would benefit from treatment (11). Limitations both in equipment, human, and financial resources cause health systems to be wary of futile care due to the high costs that naturally affect health systems, insurance companies and families (12). 

Along with these challenges, nurses’ frequent contact with futile care leads to added moral distress. When faced with such situations, nurses feel unable to provide care for their patients and this endangers their ethical principles. Nurses in one study stated that supporting end-of-life patients and providing futile care cause them to experience negative feelings such as powerlessness, hopelessness, anger, distress and guilt (13). 

Ferrell believes that nurses confront a challenge both in the case of frequent, expanded and futile care, and lack thereof (7). Nurses who have to provide futile care are faced with a dilemma: they know that the care is not effective but they must offer it. Poor quality and futile care are among moral distress factors (14). If nurses feel that treatments are not only useless but may also increase the patients’ pain and affect their good death, they will be morally distressed (7). 

Cases where the nurse has a prospect of failure, holds views opposing to those of the treatment team, and occupies a minor role within the system, are challenges to the nurse and determinants of futile care (14). 

Studies have shown that futile care has different effects on nurses. Meltzer and Huckabay found that futile care is related to moral distress, tiredness, and exhaustion (8). Additionally, observing and providing futile care puts nurses in distress and opposition (15). A survey of intensive care unit nurses showed that when treatment measures are replaced by palliative care, moral distress will be experienced (8, 16, 17). Rice et al. also considered futile care a factor for increasing moral distress (18), and Mobley et al. mentioned futile care in intensive care units as a significant source of moral distress (9).

Studies conducted in Iran indicate that moral distress is a common phenomenon (19-21), although little research has been conducted on its relationship with futile care. Global research shows that futile care is an important cause of moral distress in nurses (15-17). These issues can potentially have negative effects on nurses, patients and health systems, and impede the achievement of health objectives. Therefore, the present study was conducted to determine the relationship between moral distress and perception of futile care in intensive care nurses.

## Method

This was a cross-sectional, correlational study performed in 2013 aiming to investigate the relationship between moral distress and perception of futile care in nurses.

**Study Population and Sampling**

Study subjects included 300 nurses from Kerman teaching hospitals who were selected by convenience sampling from ICU, CCU, NICU, dialysis, and oncology wards of Bahonar, Shafa, and Afzalipour hospitals. Inclusion criteria were having a minimum bachelor’s degree and one year nursing experience in clinical wards of hospitals.

The study proposal was approved by the Ethics Committee of Shahid Beheshti University of Medical Sciences (Proposal Code: 1392-1-91-11892-14435. Ethical Code: 92:145) and legal permissions were obtained prior to collection of data. Study subjects were first provided with information on the questionnaire and the responses, and reminded that participation in the study was completely voluntary. Furthermore, participants were asked not to write their names on the questionnaires and informed that their personal information would be confidential. 

***Research Tool***

The applied tool was a questionnaire consisting of three parts: The first part included demographic information such as age, gender, years of service, and ward type.

The second part was Corley’s Moral Distress Scale designed by Corley et al. in 1995. The preliminary form of this questionnaire comprises 38 items (22), but in this study, the 21-item brief form developed by Corley and Hamrick in 2007 was used (23). The moral distress questionnaire was based on a 6-point Likert scale including 6 options in the intensity and 6 in the frequency dimension. The options in the intensity dimension varied between not at all (0) and very much (5), and in the frequency dimension between never (0) and frequently (5) (23). 

The third part consisted of a researcher-made 17-item questionnaire to evaluate the intensity and frequency of nurses’ perception of futile care situations and was designed based on Corley’s Moral Distress Scale and review of related texts. This questionnaire examined futile care situations with a focus on good death, pain and discomfort management, hasty withdrawal of treatment, effective communication with family members, disagreements among treatment team members, family members’ conflicts on treatment choices, and resource allocation. 

The responses to all three parts of the questionnaire were arranged based on a 6-point Likert scale. The options in the intensity dimension varied between not at all (0) and very much (5), and in the frequency dimension between never (0) and frequently (5). Corley’s Moral Distress questionnaire had already been translated into Persian and its reliability and validity had been verified. Borhani et al. had determined the validity (content validity index or CVI) of this questionnaire at 88%, and its reliability (Cronbach’s alpha) at 93% (19). The futile care questionnaire was also studied in terms of reliability and validity. Its validity (CVI) was determined by 10 experts on ethics at 82%, and its reliability (Cronbach’s alpha) was shown to be 85% using internal correlation coefficient. 

After receiving ethical codes and permission to obtain the subjects’ written consent from the affiliated hospitals, the questionnaires were distributed among the nurses, and collected by the researcher after completion. This process took 35 days. There were a total number of 335 nurses of whom only 300 agreed to participate; the other 35 nurses refused to participate. Data obtained from the questionnaires were registered in the SPSS version 16 software and analyzed using descriptive statistics (mean, standard deviation, frequency, frequency percentage) and inferential statistics (Pearson’s correlation, independent T-test, one-way ANOVA, etc.) to achieve the study objectives.

## Results

Demographic characteristics of the study subjects included age, gender, ward and number of years in service. The age of participating nurses ranged from 25 to 44 (mean age = 32, SD = 3.1). Eighty seven percent of the study subjects were female and 13 percent were male. The highest number of years in service was 25 and the lowest was 2 (mean = 14, SD = 4). The nurses’ ward types included ICU, CCU, NICU, oncology, and dialysis (Table 1).

**Table 1:** Ward type and gender of the nurses

**Ward type**	**NO**	**(%)**
**ICU**	160	(53.33)
**CCU**	53	(17.67)
**NICU**	35	(11.66)
**Oncology**	32	(10.67)
**Dialysis**	20	(6.67)
**Total**	300	(100)
**Gender**	**NO**	**(%)**
**Male**	39	(13)
**Female**	261	(87)
**Total**	300	(100)

No: frequency; (%): the relative Frequency

*Intensity and frequency of moral distress and futile care*

The results revealed a mean moral distress intensity of 3.54 (SD=0.3) and a mean moral distress frequency of 3.11 (SD=0.6), and the total intensity and frequency ranged from 0 to 5. The average intensity of nurses’ perception of futile care was 3.2 (SD=0.46) and the average frequency was 3.7 (SD=1.2). Total intensity and frequency ranged from 0 to 5.

A review of the section on moral distress revealed that the highest average of intensity and frequency pertained to the item [My busy work schedule causes my job quality to be reduced] with a score of 2.28 (SD=0.4). The lowest average of intensity and frequency pertained to [I start resuscitation even when I am sure this will postpone patient’s death] at 1.09 (SD=0.6).

In the section on futile care, the highest average of intensity and frequency pertained to [I do life saving actions in spite of knowing that this only postpones death] with a score of 3.08 (SD = 0.7). The lowest average pertained to [I follow physician orders on treatments and tests for a patient who is in the final stage of life, even if they seem unnecessary] at 1.89 (SD=0.8). 

*Relationship between moral distress and nurses’ perception of futile care*

There was a significant correlation between the mean of total moral distress and the mean of total nurses’ perception of futile care (*P *= 0.03, r = 0.4).

*Correlation between moral distress and nurses’ perception of futile care, and demographic characteristics*

There was a significant correlation between moral distress and age (*P* = 0.04, r = - 0.3), and between moral distress and number of years in service (*P* = 0.04, r = - 0.4). Additionally, there was a significant correlation between moral distress and type of ward (*P* = 0.03) (Table 2).

 A significant correlation was found between nurses’ perception of futile care and their age (*P* = 0.03, r = 0.4), and between their perception of futile care and number of years in service (*P* = 0.02, r = 0.5). Moreover, a significant correlation existed between nurses’ perception of futile care and type of ward (*P* = 0.04) (Table 2).

**Table 2:** Moral distress and futile care in terms of ward type

**Work Setting**	**Moral Distress** **mean (SD)**	**Futile Care** **mean (SD)**
**ICU**	4.25 (0.25)	3.2 (0.76)
**CCU**	3 (0.88)	2.6 (0.88)
**NICU**	3.99 (0.5)	2.83 (0.66)
**Oncology**	2.6 (0.72)	3.2 (0.78)
**Dialysis**	2.8 (0.65)	2.71 (0.98)
***P*** ** value**	(0.03)*	(0.04)*

SD: standard deviation

* (*P* value was significant)

## Discussion

The purpose of this research was to study the relationship between moral distress and nurses’ perception of futile care. Our findings showed that there was a meaningful and positive relationship between the two, which has been confirmed by other studies as well. Mobley et al. also studied moral distress and futile care in nurses, and reported a positive relationship between them. They found that moral distress and related conditions might increase nurses’ perception of futile care (9). In a study by Mealer et al. on the conditions in intensive care units, the frequent involvement of nurses in futile care conditions was identified as a source of stress and distress (24). Beckstrand et al. also considered end-of-life care as an important source of distress and stated that it may cause problems for nurses in various proportions (17). Moreover, organ donation was regarded by Rady and Johnson as the only factor that may be effective in causing moral conflict among nurses (13).

Analysis of these results confirmed that futile care is a phenomenon contributing to moral distress in nurses, while the consequences and challenges can in turn affect its intensity and frequency. In other words, in addition to the futility or inutility of the care being offered, the legal, professional, and moral aspects of this phenomenon may increase the nurses’ stress and even make it unbearable.

A review of the results showed that the highest average of intensity and frequency of moral distress pertained to [busy working hours decreased my job quality]. Similarly, McCarthy and Deady mentioned extended work pressure and shortage of work force in clinical settings as effective factors in moral distress (25). In her study on moral distress in intensive care nurses, Corley identified the work pressure in these units as the most prevalent cause of moral distress (25). In most studies, high work pressure, both from intensity and frequency aspects, has been mentioned as the most important factor contributing to moral distress. In the present study, the lowest average of frequency and intensity of distress pertained to [I start resuscitation even when I am sure this will postpone patient’s death]. Corley found that performing unnecessary actions that have no effect on a patient’s life was the main cause of moral distress (26). Jameton also believed that taking unnecessary care was related to moral distress and that it could lead to problems like burnout if it happened frequently (27). Based on these findings, moral distress induces various responses in nurses, and in different studies the causes for this phenomenon are shown to have different manifestations. 

In the section about nurses’ perception of futile care the highest average pertained to [I deliver life-saving care in spite of the fact that I know it may only postpone death]. Mobley et al. found this item to be rather significant in their study as well (9). The highest frequency of futile care pertained to [even if it seems unnecessary, I follow physician orders on treatments and tests for patients]. In their study Meltzer and Huckabay showed that when nurses frequently have to tolerate conditions that they know would involve futile care, and through which the patient is denied an easy death, the condition itself will affect their perception (8). 

Our findings showed that the average of moral distress from both intensity and frequency aspects was at the medium level, while some other studies reported high levels of moral distress among nurses (18, 28, 29). These studies identified moral distress as an important phenomenon that may have different effects on nurses and patients. We were also able to find some other studies in Iran that reported medium levels of moral distress (19, 21,30). 

The results showed that nurses’ perception of futile care was at a medium level from both frequency and intensity aspects, and that the average was higher in the former. Based on the results from other studies, nurses’ perception of futile care differed from the intensity and frequency points of view, although it was at a medium level overall (15, 31, 32). In their study, Meltzer and Huckabay reported a medium level of moral distress in connection with delivering futile care (8). Ferrell, however, found a high level of moral distress in nurses who provided futile care (7). It should be remembered that these studies have mostly examined futile care as a part of moral distress, and investigated its role in generating the latter.

There was a significant and inverse relationship between moral distress and the parameters of age and number of years in service. These results indicate a decrease in moral distress with increasing age and service years. Other studies have also revealed a significant correlation between the above-mentioned parameters (7, 19, 28). Research indicates that with increased age and years of service, nurses gain more experience and use effective defense mechanisms in facing ethical challenges, so they are less affected by the circumstances. Additionally, nurses who have been on the job for a longer time prefer to work in easier environments and avoid ethical challenges. On the other hand, at the beginning of their service, nurses are not sufficiently experienced to face ethical challenges, and are often involved in and influenced by crisis and confusion.

In assessing the relationship between moral distress and ward type, the highest level of distress was observed in ICU nurses (28, 33). Studies generally consider critical care units as having the highest degree of distress for nurses (7, 19, 28, 33), obviously due to the particularly acute conditions in these treatment settings that are associated with more complex ethical challenges. 

There was a significantly positive correlation between nurses’ perception of futile care, and age and tenure. Our findings showed that nurses’ perception of futile care increased both in terms of intensity and frequency with age and tenure. Other studies confirm the results of the current study (2, 23). One such study has been conducted by Mobley et al., who maintained that with increased age and tenure individuals become more prone to futile care and its challenges (9). Our study results are also confirmed by Meltzer and Huckabay, and also Cummings who found that increased age and consequently repeated encounters with futile care result in psychological changes in individuals that will make them more vulnerable against the phenomenon (8, 34).

Based on the current study results a significant relationship existed between futile care and ward type. The highest intensity and frequency of perception of futile care pertained to nurses working in ICU and oncology. Meltzer and Huckabay, and also Dunwoody found the prevalence and intensity of this phenomenon to be higher among the nurses in ICU than other units. They stated that ICU nurses were more prone to circumstances that lead to futile care because of their frequent contact with patients suffering from chronic conditions (8, 32). Mentioning that this phenomenon is more prevalent in ICU, Heland also declared that nurses delivering end-of-life care had a higher perception of futile care (35). 

## Conclusion

The results from the present study showed that moral distress and futile care had a meaningful and positive relationship. This means that futile care is an important source of moral distress and its dangerous consequences. Considering the nurses’ medium level of moral distress and perception of futile care more attention should be paid to strategies like teaching and applying positive adaptive mechanisms in order to reduce the effects of these phenomena on nurses. Information and education can raise nurses’ awareness of these phenomena, and thus help them develop coping strategies and decrease any potential negative consequences by acquiring effective defense mechanisms. 

One limitation of this study was that it was conducted in a specific region in Iran and on public hospital nurses. In order to reveal the other aspects of moral distress and perception of futile care in ICU nurses, further studies should be performed in other regions as well as in private and specialized hospitals.
